# Is Classroom Noise Always Bad for Children? The Contribution of Age and Selective Attention to Creative Performance in Noise

**DOI:** 10.3389/fpsyg.2019.00381

**Published:** 2019-02-26

**Authors:** Jessica Massonnié, Cathy Jane Rogers, Denis Mareschal, Natasha Z. Kirkham

**Affiliations:** Department of Psychological Sciences, Centre for Brain and Cognitive Development, Birkbeck, University of London, London, United Kingdom

**Keywords:** classroom noise, executive functions, creativity, selective attention, working memory

## Abstract

Creativity is considered an important skill in learning but little is known about the environmental factors affecting it in classroom settings. Extending adult findings, this study assessed whether moderate multi-talker noise promotes children’s creativity, and whether this is modulated by children’s age, working memory, and selective attention. Forty-four elementary school children between 5 and 11 years of age, divided into younger and older age groups, participated in this within-subjects’ study. The children completed two idea generation tasks; each participant performed the task both in silence and in moderate (64 dB) classroom noise. Selective attention skills, verbal and visuospatial working memory were assessed with behavioral tasks. Results showed that there were no conditions in which classroom noise promoted children’s creativity whilst some negative effects of noise were observed. Younger children (between 5 and 8 years of age) with low selective attention skills were especially at risk: they gave fewer ideas in the presence of noise, and these ideas were rated as less original. Children with good selective attention skills were globally protected against the effects of noise, performing, similarly, in silence and noise. Future studies about children’s specific creative strategies might help shed light on the mechanisms underlying these effects.

## Introduction

Creativity involves the construction of new ideas and products, which are considered both original (unique) and of value (in other words, appropriate, or useful; Runco, 2003). Learning and creativity are intertwined processes that can both be cultivated in the classroom (Guilford, 1967). According to Pang (2015), idea generation – the process of creating new and potentially useful ideas – can be seen as a part of learning, in that it induces a change in a person’s knowledge or behavior. This process of making new connections and transformations between different elements of knowledge can positively impact learning in many areas of the curriculum. For example, idea generation during a reading session in the classroom might involve encouraging children to imagine a brand new storyline or to suggest what might follow a particular event in a story (Pang, 2015). Similarly, in mathematics, idea generation can be used to redefine problems, or to find multiple ways to solve them (Beghetto and Kaufman, 2014; Pang, 2015). Idea generation is central to what children do at school.

Research on creativity has generally focussed on the cognitive processes and personality traits associated with creative thought (i.e., intelligence, knowledge base, risk-taking, openness to experience, motivation, etc.; Guilford, 1967; Runco, 2003; Barbot et al., 2016). Studies looking at the environmental factors supporting creative thought, in the classroom or in the workplace, tend to concentrate on social and organizational factors such as the level and type of support provided by teachers/managers, the presence of collaborative settings, or access to relevant resources (Amabile, 1982; de Souza Fleith, 2000; Fasko, 2001; Shalley and Gilson, 2004). However, little is known about the *physical* environmental factors, including noise, that are likely to influence creativity.

A recent paper by Mehta et al. (2012) explored the idea that a certain amount of ambient environmental noise might actually have a beneficial effect on creative processes. Using several canonical creative cognition tasks, in which participants had to generate multiple ideas and/or find links between words, Mehta et al. (2012) discovered that adults’ creativity was enhanced when in a moderate-noise environment (versus low noise or high noise). Dubbed the “Starbucks effect” this series of studies showed that when participants were exposed to noise of the level and type found in coffee shops (70 dB, with varying traffic and speech sounds overlapped), they gave more original answers compared to participants working in low noise (50 dB) and high noise (85 dB) conditions. This leaves open the question as to whether noise is beneficial or detrimental to creative cognition in childhood.

This question is particularly relevant to education because noise is a ubiquitous environmental factor in classroom learning. During class time, children are surrounded by conversational noise, sounds from devices such as computers or printers, as well as noise from adjacent classrooms or outside. Even when they are engaged in solo work, children are rarely in silence; they search for things in their pencil case, move their chairs and receive intermittent comments from their teacher. Noise levels can range from moderate (56dB during silent reading or testing), to high (76.8 dB during group work involving movement), with an average of 72 dB during the school day (Shield and Dockrell, 2004). If one is trying to promote creativity by encouraging discovery learning and collaboration in the classroom (de Souza Fleith, 2000; Fasko, 2001), noise levels are likely to be between 70 and 76.8 dB. These values are close to the “optimal” level of noise for creativity highlighted by Mehta et al. (2012). Crucially, Mehta et al. (2012) were keen to examine the effects of “real world” noise, and so used a mix of multi-talker voices, roadside traffic and distant construction noise in their study. This noise is similar to the type of noise experienced in classrooms, and more naturalistic than that previously used in research into noise and creativity (Martindale and Greenough, 1973; Kasof, 1997; Hillier et al., 2006).

Mehta et al. (2012) conclude that noise is not necessarily detrimental to performance. This is not an anomalous finding; Ljung et al. (2009) found that recorded multi-talker noise equally had no negative impact on reading speed, reading comprehension or maths and some have even shown that performance on comprehension and spelling tasks can be better in the presence of mixed noise (e.g., recorded babble noise, plus external noise, such as trucks and sirens) than quiet settings (Dockrell and Shield, 2006). Similarly, children exposed to 70 dB of “free play” noise performed better on a mathematics task, than those in a quieter condition (Zentall and Shaw, 1980). Slater (1968) tested children in their actual classroom, while noise ranging from 55 to 90 dB was coming from external rooms and corridors. They found no significant effect of noise on reading performance, compared to a quieter condition. Note that these effects seem to be specific to complex tasks such as reading and mathematics, and are in contrast with a negative impact of multi-talker classroom noise on sustained attention (Zentall and Shaw, 1980; Dockrell and Shield, 2006). Crucially, the impact of mixed, multi-talker classroom noise on performance differs from that of single-talker noise, or simpler babble noise, that is not mixed with other environmental sounds. These have been shown to impair children’s sustained attention (Dockrell and Shield, 2006), serial recall (Elliott, 2002; Klatte et al., 2007, 2010; Elliott and Briganti, 2012), as well as performance on sentence completion, spelling and arithmetic tasks (Dockrell and Shield, 2006, but see Kassinove, 1972).

Mehta et al. (2012) go a step further than the experiments on school performance by measuring potential mechanisms by which noise might impact creativity, that is to say, participants’ level of distractibility. In their study, the positive effects of moderate multi-talker noise on creativity was associated with a feeling of being distracted and less able to concentrate in comparison to lower levels of noise. A redirection of attention might therefore explain the effects of noise on creativity.

However, how attention is deployed in noisy situations is debated. Some theories point to a role of attentional resources in coping with noise, others posit automatic interference of attention by speech sounds, and still others say that both processes are at play (for a review, see Elliott, 2002). Classroom noise seems especially likely to capture attention. It often comprises an amalgam of noise sources (e.g., different people talking at the same time, external events such as road traffic, as well as local tools and devices); it is heterogeneous and irregular. Its lack of order might trigger “deviant effects” (i.e., a sound that stands out or deviates from the context, such as a door slamming or chair scraping). It is in such situations that attention can be captured and focus on the main task can shift (Hughes et al., 2007). Instead of being an easily ignorable background noise, classroom noise might require the redirection and control of attention. In this respect, Mehta et al. (2012)’s results are consistent with other recent findings that interrupting an on-going train of thought can lead to greater subsequent creativity (e.g., Baird et al., 2012; Wang et al., 2014).

However, creating new and useful ideas involves a great deal more than managing one’s attention. It also involves the manipulation, evaluation, and selection of information (Golden, 1975; Nusbaum and Silvia, 2011; Benedek et al., 2014; Edl et al., 2014; Kleibeuker et al., 2016). Therefore, it is possible that distraction from noise will be overwhelming for young children, for whom such skills are still developing. Older children, by contrast, might behave more like adults, benefiting from noise interference, since they might have developed sufficient attentional resources to integrate distractions into the creative process.

To date, the few developmental studies that have assessed how children’s ability to cope with noise evolves with age have focused on memory performance. Although memory performance is consistently *impaired* by speech noise, there is debate about whether such impairment diminishes with age (Elliott, 2002; Klatte et al., 2010). An underlying assumption is that, if attention plays a role in the ability to cope with noise, younger children, who have lower attentional skills (Best and Miller, 2010; Diamond, 2013), would be more impaired by noise than older children and adults. However, the evidence from these studies is limited by the fact that they have not assessed attention directly. The only studies which *have* directly assessed attentional skills and whether they modulate the impact of noise have involved adults (Sörqvist, 2010; Sörqvist et al., 2010; Elliott and Briganti, 2012).

To provide a clear account of the available evidence, we need to clarify the meaning of two cognitive processes: working memory and selective attention. Both skills are considered “executive functions” (EF), allowing us “to concentrate and pay attention, when going on automatic or relying on instinct or intuition would be ill-advised, insufficient, or impossible” (Diamond, 2013, p. 135). Working memory is the ability to both store and manipulate information that is no longer perceptually present. Selective attention represents resistance to external distractors, and is an aspect of inhibitory control (Miyake et al., 2000; Diamond, 2013). Sörqvist et al. (2010) tested how working memory mediates the impact of speech noise on adults’ reading comprehension. Their working memory test involved having participants identify and remember the three smallest numbers in a list. Overall, noise negatively impacted reading comprehension. Note that the noise in this experiment consisted of hearing someone telling a story (i.e., noise likely to evoke more semantic and phonological processing than mixed, multi-talker noise), which could explain the negative effect on the reading task. Notably though, participants with lower working memory skills were susceptible to greater interference from noise. Analyzing the different components of the working memory task, the authors noted that the mediation effect was mainly explained by the ability to suppress irrelevant numbers immediately from memory. Careful analyses of different EF components showed that it was selective attention, rather than working memory, which was the key factor. Other studies provided further evidence of this: a working memory measure, which did not require inhibiting previous mental representations, did not mediate the effect of speech on serial recall (Elliott and Briganti, 2012) or on reading comprehension (Sörqvist, 2010).

To sum up, the current study aims to better understand the cognitive mechanisms by which multi-talker classroom noise might either increase or depress children’s creativity. Specifically, it will address three questions: (1) Do elementary school children benefit from moderate amounts of classroom noise when performing an idea generation task? (2) Does the effect of noise vary depending on children’s age? (3) How is this effect modulated by attentional skills?

In the current study, a within-subject manipulation was used to assess the impact of noise on idea generation, with each child being tested in silence and noise. Unlike the between-subject design used by Mehta et al. (2012), this design allowed to control for confounding variables, such as inter-individual differences in baseline levels of creativity, when assessing the main effect of noise. In addition, this design was chosen to increase the ecological validity of the results as individual children in classrooms are exposed to varying levels of noise, depending both on time of day and the kind of activity they are doing. It is unlikely that different groups of children will only be exposed to one specific noise range.

The role of attention was assessed in two ways. First, to provide a developmental perspective, two groups of children were compared: those between 5 and 8 years old (early elementary/UK Key stage 1), and those between 8 and 11 years old (upper elementary/UK Key stage 2). Secondly, behavioral assessments of both working memory and selective attention were included. This way, it was possible to directly assess whether selective attention was the main component modulating the impact of noise on creativity, independently, or in conjunction with age.

We hypothesized that, in accordance with Mehta et al.’s (2012) results, children would give more original ideas in the moderate classroom noise condition than in silence. We expected this effect to interact with children’s level of selective attention, and consequently with age, since selective attention skills are known to vary with age (Lane and Pearson, 1982).

## Materials and Methods

### Participants

Forty-seven primary school children were tested at the University during a public engagement event called Bright Sparks. Children were invited to participate in pedagogical activities about the brain, as well as participate in research. Three children (two who were not fluent in English and one with a hearing impairment) were subsequently excluded. The final sample included 44 children, from 4.95 to 11.36 years of age. The children were split into two age groups representing lower (UK Key stage 1) and upper (UK Key stage 2) primary school. The younger group included children up to 8 years of age (*n* = 23, *M* = 6.54, *SD* = 0.95, 16 girls), whereas the older age group included children above 8 years of age (*n* = 21, *M* = 9.65, *SD* = 0.91, 7 girls). The project received ethical approval from both the Departmental and University Ethics Committees. Following an opt-in procedure, all the children gave verbal consent to participate, and written informed consent was obtained from their guardian. The study was conducted in accordance with the Declaration of Helsinki.

### Procedure

Children were tested individually over three short sessions. The presentation order of the three test sessions was randomized across children, and the children were given short breaks between each test session. The first two sessions included assessments of selective attention and visuospatial working memory, on one hand, and of verbal working memory, on the other hand. They were performed in silence. In the third session, two idea generation tasks were performed (Alternative Uses Task and Just Suppose, see section Measures below for details). Each of these tasks was performed once in silence and once in noise. The order and type of these creative tasks was fully counterbalanced across participants. The order of the noise conditions was counterbalanced in a semi-random way, to ensure there were neither two consecutive silent tasks nor two consecutive noisy tasks. In other words, all children consistently had to switch between silence and noise when tested for creativity. The noise stimulus consisted of classroom noise (including bits of conversation, movement noise and outside noise), played at 64.3 dB(A) on average (L_Aeq(5_ _min)_ = 63.1 dB(A); Range = [52.8–76.1 dB(A)]). This average noise level was deliberately slightly below the 70 dB target to allow for the additional noise created by the experimenter and the participant, who were themselves talking and manipulating objects. Testing took 1 h in total. Parents were invited to fill in a socio-demographic questionnaire while their child was being tested. In particular, socio-economic status was assessed to make sure that the younger and older children of our sample had a comparable family background.

### Measures

#### Socioeconomic Status

Two indicators of socio-economic status were used. First, parents reported their highest level of education (Hackman et al., 2015), coded on a 5-points Likert scale (1: High school; 2: Some college; 3: Undergraduate degree; 4: Some postgraduate; 5: Higher postgraduate). Secondly, postcodes were used to compute the Index of Multiple Deprivation corresponding to the family’s home. This index ranks areas from 1 (most deprived) to 32,844 (least deprived) according to seven domains: income, employment, education, health, crime, barriers to housing, and the living environment (Department for Communities and Local Government, 2015^1^; see Barnes et al., 2006 for the use of the IMD in educational research).

#### Working Memory

Verbal working memory was tested using a Backwards Digit Span task (St Clair-Thompson and Gathercole, 2006). Children had to repeat back in reverse order a list of digits spoken by the experimenter. List lengths started at two digits and there were four trials per list-length level. Children had to succeed on three trials to move on to the next level. The total number of correct trials was recorded. Visuospatial working memory was assessed using a computerized variant of the Corsi block task (Berch et al., 1998): the frog matrices task programmed with Matlab 9.1.0. Participants saw a display of 9 lily pads (3 × 3, see Appendix A). They had to remember the movements of a frog, jumping on the lily pads, and to click on them in reversed order (also see Morales et al., 2013, for the same task design, but using a forward recall procedure). List lengths started at two. That is to say, the frog started from a given lily pad, and jumped twice. It stayed on the final lily pad and children had to click on the previous two lily pads, starting with the most recent. There were four trials per list-length level, children having to succeed on three trials before moving on to the next level.

#### Selective Attention

Selective attention was tested using child-friendly Stroop and Flanker tasks. In the nonverbal Stroop task, programmed with Matlab 9.1.0, pairs of animals (e.g., lion and rabbit) were presented in varying sizes (Catale and Meulemans, 2009). Participants had to indicate which was the biggest animal *in real life*, an answer which corresponded to the biggest picture in congruent trials and to the smaller picture in incongruent trials (see Appendix B). In other words, children had to inhibit the perceptual characteristics of the stimuli, in order to answer according to the animals’ real relative size. There were 72 trials (50% were congruent). Trials terminated after 3000 ms. The Flanker task was adapted from Rueda et al. (2004) and programmed with Gorilla.sc^2^. Children saw a row of five fish, and had to indicate the direction the middle fish was swimming (either to the left or right). The surrounding fish were either pointing in the same direction (congruent trials) or in the opposite direction (incongruent trials). There were 96 trials (50% were congruent), and the direction of the middle fish varied randomly between left and right. Four types of trials were therefore presented (see some examples in Appendix C): all the fish pointing to the right (25%), all the fish pointing to the left (25%), middle fish pointing to the right and flanking fish to the left (25%), middle fish pointing to the left and flanking fish to the right (25%). There was no timeout within the task. However, to ensure that reaction time limits would be equivalent to that of the Stroop task, trials for which children took longer than 3000 ms to answer were excluded. Finally, for both the Flanker and Stroop tasks, RTs under 200 ms (being too short to allow perception of the stimulus) were excluded, as well as RTs above 3 standard deviations from the mean of each subject (to prevent extreme values from influencing the results).

#### Idea Generation

The Alternative Uses Task (AUT) was used to compare results with those reported in Mehta et al. (2012)’s study. Furthermore, to evaluate the generalizability of the findings, the Just Suppose test, from Torrance (2016) was also used. In the AUT, children had to come up with as many interesting and unusual uses as they could for two everyday objects – a plastic bottle and a pencil – within 3 min. They were asked to use their imagination to come up with new ideas and to go beyond the uses they had seen or heard before. The exact instructions are provided in Appendix D. In the Just Suppose task, children were presented with two imaginary situations. For the exact instructions, see Torrance (2016). After having heard each scenario, children were asked to suppose that the situation really happened, and were prompted to think about all the other things which might happen because of it, within 5 min. The two idea generation tasks were scored according to two indicators: fluency and originality. Fluency scores correspond to the total number of ideas given by a participant; all answers were counted, except answers that were an exact repetition of the instruction – e.g., for the AUT, saying that a pencil could be used to draw or to write. Elaborations such as “drawing a flower,” “drawing a house” were counted, since they were not an exact repetition of the instructions. Finally, responses that were too broad to represent a specific idea (e.g., “you can use it to make things”) were also removed. Interrater reliability, calculated on 25% of the sample, was high for the AUT (α = 1 for both objects) as well as for the Just Suppose task (α = 0.99 for both scenarios). Originality scores were calculated for each idea that contributed to the fluency score (that is to say, repetitions were also excluded for originality scoring). We followed the scoring method provided by Torrance (2016) to score the Just Suppose task. Interrater agreement was high (α_strings_ = 0.89, α_fog_ = 0.69). As in Mehta et al. (2012), originality ratings for the AUT were made by four external raters, following a “Consensual Assessment Technique” (Amabile, 1982). Using a scale from 1 (not at all creative) to 5 (highly creative), raters were instructed to take into account their “sense of originality and inventiveness of each response, in one holistic measure.” The participants’ scores were averaged for each answer. Note that this method broadly includes a rating of appropriateness in the concept of “inventiveness” though we did not want to over-emphasize that dimension since this would mean projecting adults’ judgments of utility on children’s ideas and some ideas can be meaningful to children in ways that differ from adults’ standards (Runco, 2003). Our method reflects only one way to score the AUT. The frequency method is also widely used, but revealed several limitations when we tried to apply it. This method involves compiling a list of all the answers provided by the participants, and selecting a threshold below which ideas can be considered “unusual.” For example, an idea that is given less than 5% of the time could be given a point for originality, and an idea that is given less than 1% of the time 2 points. Using this method raised two major issues. First, compiling a list of ideas and selecting which ones were “unique” was difficult, given that every answer was worded slightly differently. Interrater agreement was hard to reach. Furthermore, choosing if two similar yet different ideas (e.g., “drawing a house” and “drawing a house invaded by zombies”) should be considered “unique” seemed to reflect a process of categorization that is more characteristic of flexibility processes (the capacity to give different categories of ideas), than of originality *per se*. Given the high level of interrater reliability that was achieved using the external raters method (α_pencil_ = 0.80, α_bottle_ = 0.82), this widely-used scoring procedure was deemed preferable.

## Results

The raw data supporting the conclusions of this manuscript will be made available by the authors to any qualified researcher. Analyses of variance were performed using SPSS 23. Bayesian factors were computed with JASP 0.9.0.1.

### Pre-processing of the Selective Attention Tasks

Accuracy (the proportion of correct trials) was at ceiling for both the Flanker (*M*_congruent_ = 95.28%; *M*_incongruent_ = 92.15%) and Stroop (*M*_congruent_ = 95.02%; *M*_incongruent_ = 92.34%) tasks. Therefore, reaction times for correct answers (in both the congruent and incongruent conditions) were retained as the core measure of selective attention (see Table 1 for the descriptive statistics). Analyses of variance, with Congruency as a within-subject factor (two levels: Congruent vs. Incongruent), and Age (two levels: Young vs. Old) as a between-subject factor were carried out.

**Table 1:** Reaction times for congruent and incongruent trials at the Flanker and Stroop tasks, per age group.

	Flanker		Stroop	
		
	Congruent	Incongruent	Congruent	Incongruent
Young	987.79	1041.63	1064.90	1170.69
Old	797.71	831.03	862.05	913.36
Full sample	897.27	941.35	968.31	1048.15


For the Flanker task, reaction times were significantly longer for incongruent than congruent trials [*F*(1, 40) = 12.36, *p* = 0.001, $\eta_{p}^{2}$ = 0.236]. There was a main effect of Age, showing that children above 8 years of age were generally faster [*F*(1, 40) = 11.71, *p* = 0.001, $\eta_{p}^{2}$ = 0.226], but there was no interaction between Age and Congruency [*F*(1, 40) = 0.685, *p* = 0.413, $\eta_{p}^{2}$ = 0.017].

For the Stroop task, RTs were also longer for incongruent than congruent trials [*F*(1, 40) = 38.45, *p* < 0.001, $\eta_{p}^{2}$ = 0.490]. As for the Flanker task, children above 8 years of age were globally faster than their younger peers, as indicated by a main effect of Age [*F*(1, 40) = 18.42, *p* < 0.001, $\eta_{p}^{2}$ = 0.315]. The effect of Age interacted with that of Congruency, the difference between congruent and incongruent trials being smaller for the older group [*F*(1, 40) = 4.62, *p* < 0.038, $\eta_{p}^{2}$ = 0.104].

For each participant, a reaction time cost score was calculated, by subtracting the mean reaction time for correct answers to the congruent trials, from the mean reaction time for correct answers to incongruent trials. Higher values indicate poorer selective attention (since it takes proportionally longer to give correct answers for incongruent trials). An outlier was detected for the Flanker task, the difference in reaction times between congruent and incongruent trials being more than three standard deviations above the mean of the sample. This data point was subsequently excluded from the analyses on the Flanker task.

### Group Differences

There was no significant difference in socio-economic status between the two age groups, as revealed by a Chi-Square test carried out on the parental education measure [*χ^2^*(4) = 1.511, *p* = 0.825], and by an independent sample *T*-test performed on the Index of Multiple Deprivation (IMD) [*t*(35) = 0.34, *p* = 0.737]. Overall, parental education was relatively high: 47.7% of the parents had achieved a postgraduate level of education and 20.5% of them achieved an undergraduate level of education. Only 4.5% stopped at a college level, and 2.3% at a secondary school level of education. The median for the Index of Multiple Deprivation was 19,040, and ranged from 641 (indicating that some families came from the 10% most deprived areas of the United Kingdom), to 32,832 (10% least deprived areas). However, not all parents completed the questionnaire. Parental education and IMD data were only available for 33 (75%) and 37 (84%) children, respectively.

**Table 2:** Executive functions scores per age group.

	Younger children			Older children				
				
	*n*	*M*	*SD*	*n*	*M*	*SD*	*Indep. Sample T-test*	*BF_10_*
VWM	23	7.35	2.55	20	9.75	2.07	*t*(41) = -3.35, *p* = 0.002	19.95
VSWM	20	5.15	3.69	17	9.88	3.79	*t*(35) = -3.84, *p* < 0.001	55.69
Flanker	21	39.64	77.42	20	33.33	48.36	*t*(39) = 0.31, *p* = 0.757	0.32
Stroop	22	105.78	89.62	20	51.31	72.64	*t*(40) = 2.15, *p* = 0.038	1.83


*VWM, Verbal Working Memory; VSWM, Visuospatial Working Memory.*

Table 2 reports the means and standard deviations for each executive function measure per Age group, as well as the results of independent sample *T*-tests comparing the two groups. Missing data for some tests is due to children’s desire to stop, or programming errors (in the computerized visuospatial working memory task). For all the *T*-tests, the assumption of equality of variance between the two groups was tested with the Levene’s Test. No violations were identified, with all *p*-values above 0.281. Similarly, distributions were checked to verify the assumption of normality. Only the distributions for the Flanker task significantly departed from normality (for the younger group, Shapiro-Wilk *W* = 0.859, *p* = 0.006; for the older group, *W* = 0.873, *p* = 0.013). Results indicated that younger children had lower verbal and visuospatial working memory, and (in line with the analyses presented in section Pre-processing of the Selective Attention Tasks) higher Congruency costs at the Stroop task, indicating lower selective attention.

Bayes Factors in favor of the alternative hypothesis (noted BF_10_) were also calculated. The alternative hypothesis states that there is a difference between the two age groups. Tests were double-sided to mimic the *T*-tests. Bayes factors offer the advantage of quantifying evidence in favor of the alternative hypothesis in a more continuous fashion than the *p*-value. The magnitude of the evidence is presented as an odds-ratio (Quintana and Williams, 2018). Here, the Bayes Factor for the verbal working memory test indicated that the observed data was 19.95 more likely under the alternative hypotheses than the null. This could be considered as strong evidence for a difference between the two age groups (Wagenmakers et al., 2018a). Similarly, the Bayes factor for the visuospatial working memory brings confidence in the *T*-tests result, providing very strong evidence in favor of the alternative hypotheses. However, the age difference at the Stroop task, as assessed by the Bayes factor, can be considered inconclusive.

### The Impact of Classroom Noise on Children’s Creativity

Next, we assessed the impact of noise on creativity scores, and its potential interaction with Age. A MANOVA was run for each of the four creativity scores (AUT Fluency and Originality, Just Suppose Fluency, and Originality). The dependent variables (repeated measures) were the scores in silence and noise. The three counterbalancing factors and Age group were entered as independent, between-subject variables. Bayes factors were also computed. They were extracted from the analysis of effect of Bayesian Repeated Measures ANOVAs, using the same variables as the classical models. We used the default prior included in JASP 0.9.0.1. Bayes factors not only offer the advantage of providing a more continuous representation of the evidence in favor of the alternative hypothesis, they also allow us to weight the evidence for the null hypothesis. In other words, they can be used to assess the evidence of an effect (evidence for the alternative hypothesis, noted BF_10_), and the evidence for the *absence* of an effect (evidence for the null hypothesis, noted BF_01_). Indicative thresholds to measure the strength of the evidence range from 3 (moderate evidence) to 100 (very strong evidence). Numbers between 10 and 30 represent strong evidence. More information on Bayesian models and the corresponding procedures can be found in Quintana and Williams (2018) and Wagenmakers et al. (2018a,b).

Since the within-subject difference between creativity scores obtained in silence and noise was the focus of these analyses, for both types of analyses, data points for which this difference was three standard deviations from the mean were excluded from the analyses. This corresponded to a maximum of one child being excluded per creativity test.

**Table 3:** Scores at the AUT for the younger and older children, in silence and noise.

	Younger Children				Older Children			
		
	Min	Max	*M*	*SD*	Min	Max	*M*	*SD*
**FLUENCY**								
Silence	2	21	9	4.84	3	17	8.29	3.65
Noise	1	19	8.91	4.98	4	20	7.95	4.24
**ORIGINALITY**								
Silence	1.71	3.50	2.59	0.52	1.42	3.78	2.92	0.55
Noise	1	3.50	2.36	0.66	2.02	4.25	2.97	0.55


**Table 4:** Scores at the Just Suppose task for the younger and older children, in silence and noise.

	Younger children				Older children			
		
	Min	Max	*M*	*SD*	Min	Max	*M*	*SD*
**FLUENCY**								
Silence	1	23	9.82	5.67	3	20	11.33	4.54
Noise	2	19	8.23	4.77	2	20	11.19	5.09
**ORIGINALITY**								
Silence	1	20	7.36	4.82	2	18	8.57	4.02
Noise	0	18	5.55	4.45	1	18	8.86	4.49


Descriptive statistics are reported in Tables 3, 4.

#### Alternative Uses Task

##### Fluency scores

There was no main effect of Noise on the fluency scores in the Alternative Uses Task [*F*(1, 38) = 0.21, *p* = 0.651, $\eta_{p}^{2}$ = 0.005]. The Bayes Factor indicates that the null hypothesis (of no difference between silent and noisy sessions) is 12.66 times more likely that the alternative hypothesis stating that there is a difference. There was no main effect of Age on the fluency scores [*F*(1, 38) = 1.37, *p* = 0.249, $\eta_{p}^{2}$ = 0.035, BF_01_ = 2.80]. Finally, the effect of Noise did not interact with Age [*F*(1, 38) = 0.02, *p* = 0.887, $\eta_{p}^{2}$ = 0.001, BF_01_ = 12.20].

##### Originality scores

For the originality scores, traditional MANOVAs indicated no main effect of Noise [*F*(1, 38) = 0.94, *p* = 0.338, $\eta_{p}^{2}$ = 0.024, BF_01_ = 4.48]. There was a main effect of Age [*F*(1, 38) = 9.11, *p* = 0.005, $\eta_{p}^{2}$ = 0.193], showing that older children gave more original answers than their younger counterparts. This was supported by a Bayesian Factor indicating that the alternative hypothesis was 9.31 more likely than the null hypothesis. Although the effect of Noise significantly interacted with Age [*F*(1, 38) = 5.05, *p* = 0.030, $\eta_{p}^{2}$ = 0.117], this was not strongly supported by Bayesian analyses (BF_10_ = 1.38). Follow-up repeated measures *T*-tests indicated that the difference in performance between silent and noisy sessions was neither significant for the younger children [*t*(21) = 1.76, *p* = 0.092, BF_01_ = 1.20], nor for the older ones [*t*(20) = -0.43, *p* = 0.672, BF_01_ = 4.04].

#### Just Suppose

##### Fluency scores

There was no main effect of Noise [*F*(1, 38) = 2.97, *p* = 0.093, $\eta_{p}^{2}$ = 0.073, BF_01_ = 3.40] and no main effect of Age [*F*(1, 38) = 2.65, *p* = 0.112, $\eta_{p}^{2}$ = 0.065, BF_01_ = 1.24] on the fluency scores at the Just Suppose task. Furthermore, the interaction between Noise and Age was not significant [*F*(1, 38) = 3.13, *p* = 0.085, $\eta_{p}^{2}$ = 0.076, BF_01_ = 2.10].

##### Originality scores

Regarding Originality scores at the Just Suppose test, there was no main effect of noise [*F*(1, 38) = 2.67, *p* = 0.111, $\eta_{p}^{2}$ = 0.066, BF_01_ = 1.40]. There was no main effect of Age [*F*(1, 38) = 3.165, *p* = 0.083, $\eta_{p}^{2}$ = 0.077, BF_01_ = 1.24], but an interaction between the effect of Noise and of Age [*F*(1, 38) = 4.97, *p* = 0.032, $\eta_{p}^{2}$ = 0.116]. Younger children had lower originality scores in noise compared to silence [*t*(21) = 2.24, *p* = 0.036, BF_10_ = 1.75], whereas there was no significant difference between the conditions for older children [*t*(20) = -0.46, *p* = 0.653, BF_01_ = 4.00]. Note, however, that the interaction is not strongly supported by Bayesian analyses (BF_10_ = 1.04).

### The Modulating Role of Executive Functions

Developmental differences only provide indirect evidence for the role of executive functions in coping with noise (since executive control tends to improve with age). Therefore, further analyses were carried out to investigate whether there were any two-way interactions between the effect of Noise and Executive Functions, or three-way interactions between Noise, Executive Functions and Age. For each of the four creativity measures (AUT Fluency and Originality, Just Suppose Fluency and Originality) the same variables as in section The Impact of Classroom Noise on Children’s Creativity were entered into an MANOVA, but verbal working memory, visual working memory, Stroop and Flanker performance were added as between-subject factors, in four successive models. For each executive function variable, a “low” and a “high” performance group was created, based on the median score of the sample for each test.

There were no interactions between Noise and Executive Functions, nor any three-way interaction between Noise, Executive Functions and Age for the AUT Fluency and Originality scores. However, there were two significant interactions involving Originality scores in the Just Suppose task.

**FIGURE 1:**
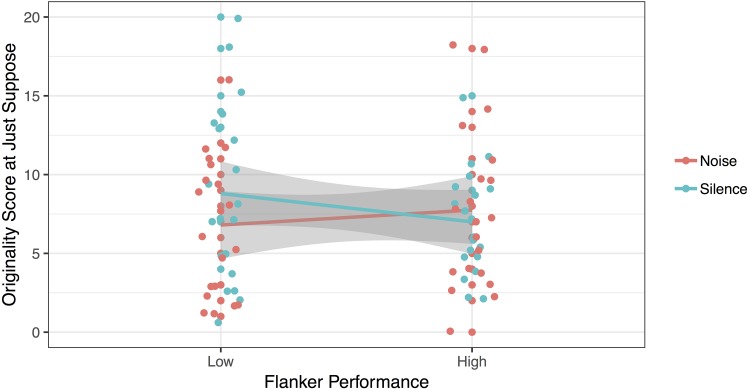
Originality of answers at the Just Suppose task, as a function of Flanker performance.

First, the impact of Noise on the Originality scores in the Just Suppose task interacted with selective attention as assessed by the Flanker task [*F*(1, 33) = 12.86, *p* < 0.001, $\eta_{p}^{2}$ = 0.280, BF_10_ = 5.57]. This interaction is depicted in Figure 1. Follow-up *T*-tests revealed that children with low selective attention gave ideas that were less original in noise (*M* = 6.80), compared to silence [*M* = 8.80; *t*(19) = 2.67, *p* = 0.015, BF_10_ = 3.60]. In other words, children who were sensitive to incongruent distractors at the Flanker task (*M_RTcost_* = 84.25 ms) were also impeded by noise when they performed their creative task. In contrast, there was no significant difference in performance between the silent (*M* = 7.00) and noisy (*M* = 7.75) sessions for children with high selective attention skills [*t*(19) = -1.097, *p* = 0.287, BF_01_ = 2.54]. Interestingly, these children were either more resistant to interference on incongruent trials in the Flanker task, or were faster at incongruent trials (*M_RTcost_* = -8.07 ms). In other words, the children who did not experience the expected Flanker interference also did not experience interference from noise.

**FIGURE 2:**
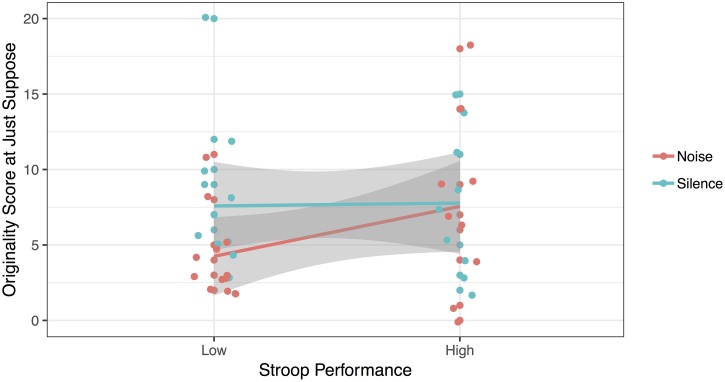
Originality of answers at the Just Suppose task, as a function of Stroop Performance, for the younger children.

**FIGURE 3:**
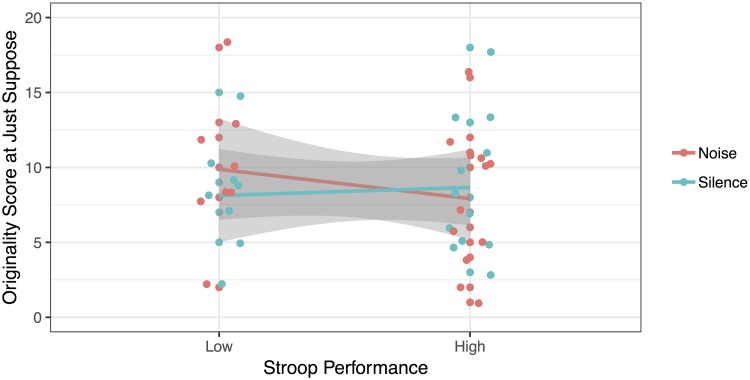
Originality of answers at the Just Suppose task, as a function of Stroop performance, for the older children.

In addition to this two-way interaction, analyses revealed a three-way interaction between Noise, Age and the second measure of selective attention, the Stroop task [*F*(1, 34) = 9.59, *p* = 0.004, $\eta_{p}^{2}$ = 0.220, BF_10_ = 1.77]. Follow-up *T*-tests revealed that young children with low selective attention (*M*_RTcost_ = 166.91 ms) gave more original answers in silence (*M* = 7.58) compared to noise [*M* = 4.25; *t*(11) = 4.318, *p* = 0.001, BF_10_ = 33.89]. This effect was very strong. In contrast, there was no significant difference in originality scores between the noisy and silent sessions for the young children with high selective attention [*M*_RTcost_ = 25.39 ms; *M*_Silence_ = 7.78; *M*_Noise_ = 7.56; *t*(8) = 0.149, *p* = 0.885, BF_01_ = 3.08] and for the older children with low selective attention [*M*_RTcost_ = 122.41 ms; *M*_Silence_ = 8.13; *M*_Noise_ = 9.88; *t*(7) = -2.084, *p* = 0.076, BF_01_ = 0.72] and high selective attention [*M*_RTcost_ = 4.08 ms; *M*_Silence_ = 8.67; *M*_Noise_ = 7.92; *t*(11) = 0.888, *p* = 0.394, BF_01_ = 2.50]. These results are represented in Figure 2, 3.

A similar three-way interaction between Noise, Age and Stroop performance emerged for Fluency scores at the Just Suppose task [*F*(1, 34) = 4.35, *p* = 0.045, $\eta_{p}^{2}$ = 0.113, BF_10_ = 0.38]. Follow-up *T*-tests parallel the previous results on originality scores. Young children with low selective attention gave more ideas in silence (*M* = 9.67) compared to noise [*M* = 7.08; *t*(11) = 2.416, *p* = 0.034, BF_10_ = 2.22]. On the contrary, there was no significant difference in fluency scores between the noisy and silent sessions for the young children with high selective attention [*M*_Silence_ = 11; *M*_Noise_ = 10; *t*(8) = 0.832, *p* = 0.430, BF_01_ = 2.34] and for the older children with low [*M*_Silence_ = 10.50; *M*_Noise_ = 12; *t*(7) = -1.620, *p* = 0.149, BF_01_ = 1.16] and high [*M*_Silence_ = 11.17; *M*_Noise_ = 10.33; *t*(11) = 1.034, *p* = 0.323, BF_01_ = 2.23] selective attention.

## Discussion

To our knowledge, this is the first developmental study assessing the impact of classroom noise on children’s creativity. Two age groups, corresponding to early elementary school (5–8 years of age) and late elementary school (8–11 years of age) performed two idea generation tasks in silence and in noise. The creation of two age groups was based on the assumption that older children would have better attentional and working memory skills.

Analyses of our selected tasks showed that this difference was more striking for our working memory than our selective attention tasks (Flanker and Stroop). In the Flanker task, the younger children did not show a larger difference between congruent and incongruent trials than the older children. This is consistent with Rueda et al. (2004) who found no effect of age on reaction times using a similar task with 6–9 years old. With regards to the Stroop task, analyses revealed that interference effects were greater for younger children. However, Bayesian analyses did not provide strong evidence for this difference. Results from Catale and Meulemans (2009), who used a similar Stroop task, indicate that the presence of a significant age effect might depend on the specific way age groups are created and compared, and might require stronger statistical power. On the contrary, age differences in the two working memory tasks were both supported by traditional *T*-test analyses as well as by Bayes Factors analyses, giving strong evidence for the alternative hypothesis. Given that different components of executive functions demonstrate different developmental trajectories (Anderson, 2002) these contrasting findings are not unexpected.

Looking at age differences for the creativity scores, we found that, in the AUT, older children gave ideas that were rated as more original. This effect was supported by strong evidence from Bayes Factor analyses. Both working memory and selective attention are thought to play a role in the generation of original ideas (Nusbaum and Silvia, 2011; Beaty and Silvia, 2012; Benedek et al., 2014; Kleibeuker et al., 2016). Therefore, older children, with more developed executive function skills might be able to give more original ideas. Other factors, such as knowledge and intelligence development, might also play a role (Sternberg and O’Hara, 1999; Sternberg, 2006).

In line with Mehta et al. (2012), we expected noise to specifically and positively impact originality scores, but we also hypothesized that this might depend on children’s age (i.e., noise might be too overwhelming for children in early elementary school). Results revealed that the effect of noise on originality scores in the AUT and Just Suppose tasks significantly interacted with age. Older children performed, similarly, in both conditions, in both tests. The younger children gave fewer original ideas in noise than in silence for the Just Suppose task, but performed, similarly, in both conditions in the AUT. To sum up, we can conclude that, contrary to our expectations, older children did not benefit from noise when performing an idea generation task.

A direct assessment of working memory and selective attention made it possible to test whether these EF components modulated the impact of noise on creativity in our sample. Similarly, to Sörqvist et al. (2010)’s results with adults, selective attention, but not working memory, did interact with the effect of noise. Children who experienced more interference in the Flanker task (i.e., those with poorer selective attention) gave fewer original ideas in noise than in silence in the Just Suppose task. For those who experienced less interference (i.e., those with better selective attention) there was no significant difference between the two conditions. In other words, the ability to resist interference from visual distractors went along with being less impeded by noise when generating new ideas. This is the first study giving insight on such interindividual differences in children.

Furthermore, a three-way interaction between the effect of Noise, Stroop performance, and Age emerged in predicting originality scores in the Just Suppose task. Children who did not show interference in the Stroop task appeared also to be immune to the effects of noise. Children who *did* experience Stroop interference were differentially affected according to their age. The younger children performed better in silence, whereas the older children performed, similarly, in the two conditions. Results therefore reveal that children in their early elementary school years, with low selective attention skills, might be especially sensitive to the effect of noise when performing an idea generation task. This was strongly supported by Bayesian analyses. Note that our follow-up analyses on older pupils were not as strongly supported by Bayesian factors. However, contrary to what we expected, older children (in their late elementary school years), did not perform significantly better in the presence of moderate background noise, whatever their level of selective attention. It is possible that Mehta et al. (2012)’s findings do not replicate on children.

However, we should note that our sample size was relatively small, resulting in age groups that were pretty broad. A higher number of participants (e.g., 20–30 children per school year) would allow for a more fine-grained understanding of developmental effects. A focus on the early elementary school years might be especially relevant. Indeed, noise levels in classrooms tend to decrease as children get older (Picard and Bradley, 2001), but the present findings suggest that younger children are actually more impaired by noise. We might therefore want to consider ways to reduce noise disturbance, or to develop protecting factors against noise, especially in the younger age groups. School interventions have been shown to improve children’s executive functions, and to be especially effective for children who initially start with lower levels of performance (Diamond and Lee, 2011; Diamond, 2014). More specifically, selective attention as assessed by the Flanker task has been shown to be improved following meditation training on an adult population (Tang et al., 2007).

Furthermore, acquiring a better understanding of the specific strategies children use in idea generation tasks would help clarify the mechanisms noise acts upon. Mehta et al. (2012) proposed “abstractness” as the main factor leading to creative answers. But children might also use visual strategies, or networks of associations in semantic memory (Gilhooly et al., 2007). Identifying these processes and their disruption might help to understand why younger children with low selective attention lack the cognitive resources to deal with *both* the task in hand *and* the noise.

Finally, to enhance the ecological validity of our findings, fluctuations in noise type and levels could be measured as they occur in the classroom, during creative activities (i.e., art lessons) and put into perspective with children’s creations and reported thinking processes. Mixed-methods would be especially relevant.

## Conclusion

To sum up, this is the first study attempting to assess the impact of moderate classroom noise on children’s ability to generate new ideas, and the role that selective attention plays. Analyses revealed that young children with low selective attention skills might be especially vulnerable to the effect of noise: they gave fewer ideas in the presence of noise, and these ideas were rated as less original. Having good selective attention skills might be globally protective, whatever children’s age. Future studies about children’s specific creative strategies might help shed light on the mechanisms underlying these effects.

## Author Contributions

JM, CR, DM, and NK contributed conception and design of the study. JM and CR collected the data. JM performed the statistical analyses and wrote the manuscript. All authors contributed to manuscript revision, read and approved the submitted version.

## Conflict of Interest Statement

The authors declare that the research was conducted in the absence of any commercial or financial relationships that could be construed as a potential conflict of interest.

## Footnotes

1 http://dclgapps.communities.gov.uk/imd/idmap.html

2 https://gorilla.sc/

## References

[B1] Amabile T. M. (1982). Social psychology of creativity: a consensual assessment technique. *J. Personal. Soc. Psychol.* 43 997–1013. 10.1037/0022-3514.43.5.997 16060417

[B2] Anderson P. (2002). Assessment and development of executive function (EF) during childhood. *Child Neuropsychol.* 8 71–82. 10.1076/chin.8.2.71.8724 12638061

[B3] Baird B. Smallwood J. Mrazek M. D. Kam J. W. Franklin M. S. Schooler J. W. (2012). Inspired by distraction mind wandering facilitates creative incubation. *Psychol. Sci.* 23 1117–1122. 10.1177/0956797612446024 22941876

[B4] Barbot B. Lubart T. I. Besançon M. (2016). “Peaks, slumps, and bumps”: individual differences in the development of creativity in children and adolescents. *New Dir. Child Adolesc. Dev.* 2016 33–45. 10.1002/cad.20152 26994723

[B5] Barnes J. Belsky J. Broomfield K. A. Melhuish E. the National Evaluation of Sure Start [NESS] Research Team (2006). Neighbourhood deprivation, school disorder and academic achievement in primary schools in deprived communities in England. *Int. J. Behav. Dev.* 30 127–136. 10.1177/0165025406063585

[B6] Beaty R. E. Silvia P. J. (2012). Why do ideas get more creative across time? An executive interpretation of the serial order effect in divergent thinking tasks. *Psychol. Aesthet. Creat. Arts* 6 309–319. 10.1037/a0029171

[B7] Beghetto R. A. Kaufman J. C. (2014). Classroom contexts for creativity. *High Ability Stud.* 25 53–69. 10.1080/13598139.2014.905247 16123462

[B8] Benedek M. Jauk E. Sommer M. Arendasy M. Neubauer A. C. (2014). Intelligence, creativity, and cognitive control: the common and differential involvement of executive functions in intelligence and creativity. *Intelligence* 46 73–83. 10.1016/j.intell.2014.05.007 25278640 PMC4175011

[B9] Berch D. B. Krikorian R. Huha E. M. (1998). The corsi block-tapping task: methodological and theoretical considerations. *Brain Cogn.* 38 317–338. 10.1006/brcg.1998.1039 9841789

[B10] Best J. R. Miller P. H. (2010). A developmental perspective on executive function. *Child Dev.* 81 1641–1660. 10.1111/j.1467-8624.2010.01499.x 21077853 PMC3058827

[B11] Catale C. Meulemans T. (2009). The Real Animal Size Test (RAST) a new measure of inhibitory control for young children. *Eur. J. Psychol. Assess.* 25 83–91. 10.1027/1015-5759.25.2.83

[B12] de Souza Fleith D. (2000). Teacher and student perceptions of creativity in the classroom environment. *Roeper Rev.* 22 148–153. 10.1080/02783190009554022

[B13] Department for Communities, and Local Government (2015). *The English Index of Multiple Deprivation (IMD) 2015 – Guidance.* Available at: https://assets.publishing.service.gov.uk/government/uploads/system/uploads/attachment_data/file/464430/English_Index_of_Multiple_Deprivation_2015_-_Guidance.pdf.

[B14] Diamond A. (2013). Executive functions. *Ann. Rev. Psychol.* 64 135–168. 10.1146/annurev-psych-113011-143750 23020641 PMC4084861

[B15] Diamond A. (2014). Want to optimize executive functions and academic outcomes?: simple, just nourish the human spirit. *Minn. Symp. Child Psychol. Ser.* 37 205–232. 25360055 PMC4210770

[B16] Diamond A. Lee K. (2011). Interventions shown to aid executive function development in children 4 to 12 years old. *Science* 333 959–964. 10.1126/science.1204529 21852486 PMC3159917

[B17] Dockrell J. E. Shield B. M. (2006). Acoustical barriers in classrooms: the impact of noise on performance in the classroom. *Br. Educ. Res. J.* 32 509–525. 10.1080/01411920600635494

[B18] Edl S. Benedek M. Papousek I. Weiss E. M. Fink A. (2014). Creativity and the stroop interference effect. *Personal. Individ. Differ.* 69 38–42. 10.1016/j.paid.2014.05.009

[B19] Elliott E. M. (2002). The irrelevant-speech effect and children: theoretical implications of developmental change. *Mem. Cogn.* 30 478–487. 10.3758/BF03194948 12061768

[B20] Elliott E. M. Briganti A. M. (2012). Investigating the role of attentional resources in the irrelevant speech effect. *Acta Psychol.* 140 64–74. 10.1016/j.actpsy.2012.02.009 22459560

[B21] Fasko D. (2001). Education and creativity. *Creat. Res. J.* 13 317–327. 10.1207/S15326934CRJ1334_09

[B22] Gilhooly K. J. Fioratou E. Anthony S. H. Wynn V. (2007). Divergent thinking: strategies and executive involvement in generating novel uses for familiar objects. *Br. J. Psychol.* 98 611–625. 10.1111/j.2044-8295.2007.tb00467.x 17535464

[B23] Golden C. J. (1975). The measurement of creativity by the stroop color and word test. *J. Personal. Assess.* 39 502–506. 10.1207/s15327752jpa3905_9 1185503

[B24] Guilford J. P. (1967). Creativity: yesterday, today and tomorrow. *J. Creat. Behav.* 1 3–14. 10.1002/j.2162-6057.1967.tb00002.x

[B25] Hackman D. A. Gallop R. Evans G. W. Farah M. J. (2015). Socioeconomic status and executive function: developmental trajectories and mediation. *Dev. Sci.* 18 686–702. 10.1111/desc.12246 25659838

[B26] Hillier A. Alexander J. K. Beversdorf D. Q. (2006). The effect of auditory stressors on cognitive flexibility. *Neurocase* 12 228–231. 10.1080/13554790600878887 17000592

[B27] Hughes R. Vachon F. Jones D. (2007). disruption of short-term memory by changing and deviant sounds: support for a duplex-mechanism account of auditory distraction. *J. Exp. Psychol. Learn. Mem. Cogn.* 33 1050–1061. 10.1037/0278-7393.33.6.1050 17983312

[B28] Kasof J. (1997). Creativity and breadth of attention. *Creat. Res. J.* 10 303–315. 10.1207/s15326934crj1004_2

[B29] Kassinove H. (1972). Effects of meaningful auditory stimulation on children’s scholastic performance. *J. Educ. Psychol.* 63 526–530. 10.1037/h0033747 4645340

[B30] Klatte M. Lachmann T. Schlittmeier S. Hellbrück J. (2010). The irrelevant sound effect in short-term memory: is there developmental change? *Eur. J. Cogn. Psychol.* 22 1168–1191. 10.1080/09541440903378250

[B31] Klatte M. Meis M. Sukowski H. Schick A. (2007). Effects of irrelevant speech and traffic noise on speech perception and cognitive performance in elementary school children. *Noise Health* 9 64–74. 10.4103/1463-1741.36982 18025757

[B32] Kleibeuker S. W. De Dreu C. K. W. Crone E. A. (2016). Creativity development in adolescence: insight from behavior, brain, and training studies. *New Dir. Child Adolesc. Dev.* 2016 73–84. 10.1002/cad.20148 26994726

[B33] Lane D. M. Pearson D. A. (1982). The development of selective attention. *Merrill Palmer Q.* 28 317–337.

[B34] Ljung R. Sorqvist P. Hygge S. (2009). Effects of road traffic noise and irrelevant speech on children’s reading and mathematical performance. *Noise Health* 11:194. 10.4103/1463-1741.56212 19805928

[B35] Martindale C. Greenough J. (1973). The differential effect of increased arousal on creative and intellectual performance. *J. Genet. Psychol.* 123 329–335. 10.1080/00221325.1973.10532692 4764419

[B36] Mehta R. Zhu R. J. Cheema A. (2012). Is noise always bad? Exploring the effects of ambient noise on creative cognition. *J. Consum. Res.* 39 784–799. 10.1086/665048

[B37] Miyake A. Friedman N. P. Emerson M. J. Witzki A. H. Howerter A. Wager T. D. (2000). The unity and diversity of executive functions and their contributions to complex “frontal lobe” tasks: a latent variable analysis. *Cogn. Psychol.* 41 49–100. 10.1006/cogp.1999.0734 10945922

[B38] Morales J. Calvo A. Bialystok E. (2013). Working memory development in monolingual and bilingual children. *J. Exp. Child Psychol.* 114 187–202. 10.1016/j.jecp.2012.09.002 23059128 PMC3508395

[B39] Nusbaum E. C. Silvia P. J. (2011). Are intelligence and creativity really so different?: fluid intelligence, executive processes, and strategy use in divergent thinking. *Intelligence* 39 36–45. 10.1016/j.intell.2010.11.002

[B40] Pang W. (2015). Promoting creativity in the classroom: a generative view. *Psychol. Aestheti. Creat. Arts* 9 122–127. 10.1037/aca0000009

[B41] Picard M. Bradley J. S. (2001). Revisiting speech interference in classrooms: revisando la interferencia en el habla dentro del salón de clases. *Audiology* 40 221–244. 10.3109/00206090109073117 11688542

[B42] Quintana D. S. Williams D. R. (2018). Bayesian alternatives for common null-hypothesis significance tests in psychiatry: a non-technical guide using JASP. *BMC Psychiatry* 18:178. 10.1186/s12888-018-1761-4 29879931 PMC5991426

[B43] Rueda M. R. Fan J. McCandliss B. D. Halparin J. D. Gruber D. B. Lercari L. P. (2004). Development of attentional networks in childhood. *Neuropsychologia* 42 1029–1040. 10.1016/j.neuropsychologia.2003.12.012 15093142

[B44] Runco M. A. (2003). Education for creative potential. *Scand. J. Educ. Res.* 47 317–324. 10.1080/00313830308598

[B45] Shalley C. E. Gilson L. L. (2004). What leaders need to know: a review of social and contextual factors that can foster or hinder creativity. *Leadersh. Q.* 15 33–53. 10.1016/j.leaqua.2003.12.004

[B46] Shield B. Dockrell J. E. (2004). External and internal noise surveys of London primary schools. *J. Acoust. Soc. Am.* 115 730–738. 10.1121/1.1635837 15000185

[B47] Slater B. R. (1968). Effects of noise on pupil performance. *J. Educ. Psychol.* 59 239–243. 10.1037/h0026025 5672237

[B48] Sörqvist P. (2010). Effects of aircraft noise and speech on prose memory: what role for working memory capacity? *J. Environ. Psychol.* 30 112–118. 10.1016/j.jenvp.2009.11.004

[B49] Sörqvist P. Halin N. Hygge S. (2010). Individual differences in susceptibility to the effects of speech on reading comprehension. *Appl. Cogn. Psychol.* 24 67–76. 10.1002/acp.1543 27566326

[B50] St Clair-Thompson H. L. Gathercole S. E. (2006). Executive functions and achievements in school: shifting, updating, inhibition, and working memory. *Q. J. Exp. Psychol.* 59 745–759. 10.1080/17470210500162854 16707360

[B51] Sternberg R. J. (2006). The nature of creativity. *Creat. Res. J.* 18 87–98. 10.1207/s15326934crj1801_10

[B52] Sternberg R. J. O’Hara L. (1999). “Creativity and intelligence,” in *Handbook of Creativity*, ed. Sternberg R. J. (Cambridge: Cambridge university press).

[B53] Tang Y. Y. Ma Y. Wang J. Fan Y. Feng S. Lu Q. (2007). Short- term meditation training improves attention and self-regulation. *Proc. Natl. Acad. Sci.* 104 17152–17156. 10.1073/pnas.0707678104 17940025 PMC2040428

[B54] Torrance E. P. (2016). *Torrance Tests of Creative Thinking. Scholastic Testing Service.* Princeton, NJ: Personal Press.

[B55] Wagenmakers E.-J. Love J. Marsman M. Jamil T. Ly A. Verhagen J. (2018a). Bayesian inference for psychology. part II: example applications with JASP. *Psychonom. Bull. Rev.* 25 58–76. 10.3758/s13423-017-1323-7 28685272 PMC5862926

[B56] Wagenmakers E.-J. Marsman M. Jamil T. Ly A. Verhagen J. Love J. (2018b). Bayesian inference for psychology. part I: theoretical advantages and practical ramifications. *Psychonom. Bull. Rev.* 25 35–57. 10.3758/s13423-017-1343-3 28779455 PMC5862936

[B57] Wang X. Ye S. Teo H. H. (2014). “Effects of interruptions on creative thinking,” in *Proceedings of the Thirty Fifth International Conference on Information Systems*, (Auckland: ICIS), 1–10.

[B58] Zentall S. S. Shaw J. H. (1980). Effects of classroom noise on performance and activity of second-grade hyperactive and control children. *J. Educ. Psychol.* 72 830–840. 10.1037/0022-0663.72.6.830 7204739

