# Supplementary material for: Is Classroom Noise Always Bad for Children? The Contribution of Age and Selective Attention to Creative Performance in Noise
Source: Front Psychol. 2019 Feb 26;10:381. doi: 10.3389/fpsyg.2019.00381 (PMC6399383; doi:10.3389/fpsyg.2019.00381)
Supplement: Supplementary file 1 [file Table_1.docx]

Appendix A

Stimulus for the visuospatial working memory task

This is a sample of the instructions, presenting the display of 9 lily pads as well as the frog who will make its way through this display.

Appendix B

Stimuli for the Stroop task

(a) Example of a congruent trial, as presented in the instructions

(b) Example of an incongruent trial, as presented in the instructions

Appendix C

Stimuli for the Flanker task


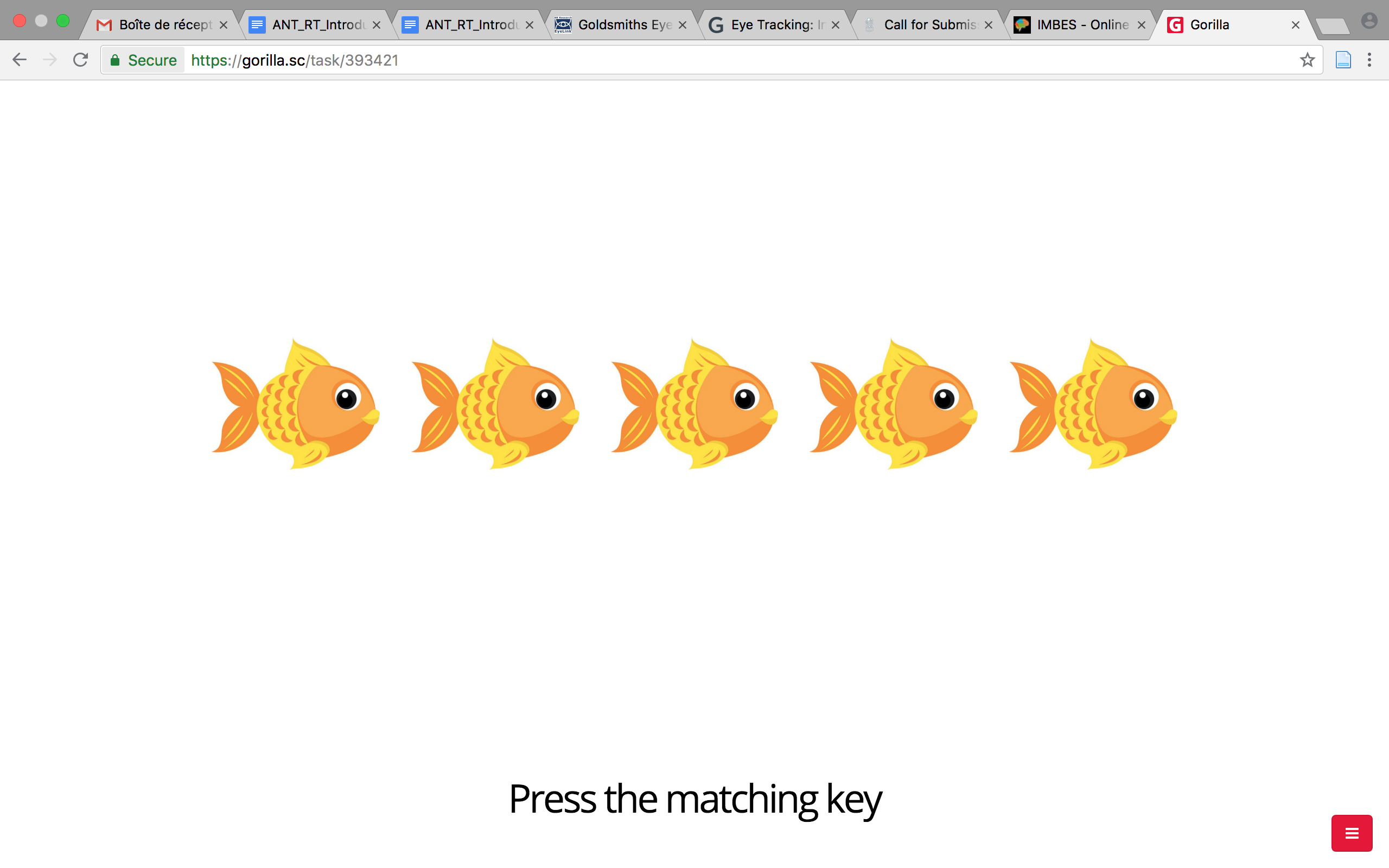


(a) Screen display for a congruent trial with all the fish pointing to the right


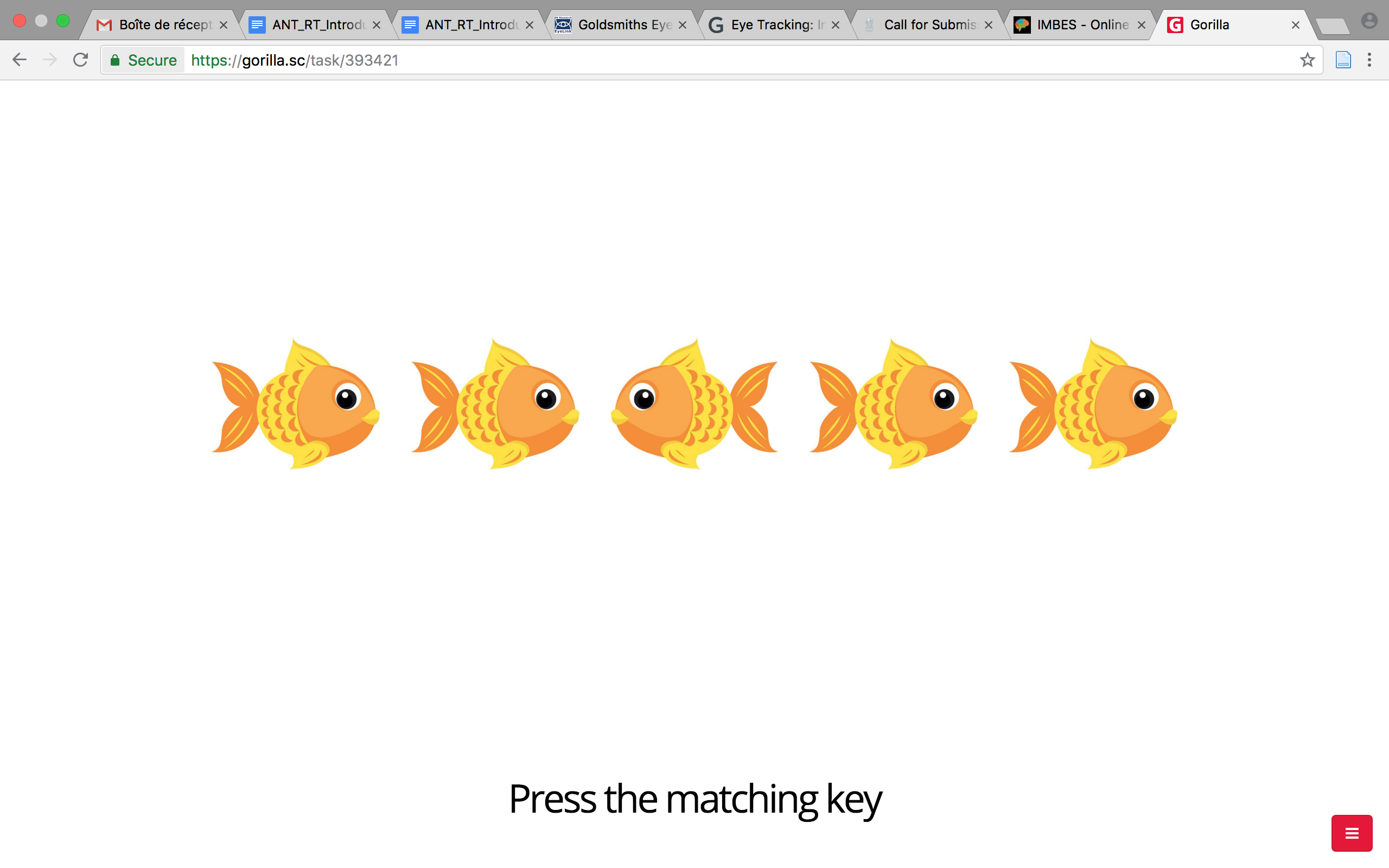


(b) Screen display for an incongruent trial with the middle fish pointing to the left and the flankers pointing to the right

Appendix D

Instructions for the Alternative Uses Task

(a) Instructions for the item ‘pencil’

Everyone knows that you can use a pencil for drawing or writing, but a pencil could also have lots and lots of other interesting and unusual uses. We want you to think of some. Don’t just think about uses you might have seen or heard before, use your imagination to try to come up with new ideas.

The great thing is there are no right or wrong answers. Try to come up with as many unusual ideas as you can.

If you say your answers out loud, I will write them down for you. OK? Let’s go!

(b) Instructions for the item ‘bottle’

Everyone knows that you can use a plastic bottle for drinking from, but a plastic bottle could also have lots and lots of other interesting and unusual uses. We want you to think of some. Don’t just think about uses you might have seen or heard before, use your imagination to try to come up with new ideas.

The great thing is there are no right or wrong answers. Try to come up with as many unusual ideas as you can.

If you say your answers out loud, I will write them down for you. OK? Let’s go!
